# Strengthening public understanding and prevention of dengue in Malaysia: insights and recommendations based on knowledge, attitudes, and practices toward vector control and vaccination

**DOI:** 10.3389/fpubh.2026.1807183

**Published:** 2026-08-20

**Authors:** Asrul Akmal Shafie, Zulkifli Ismail, Ken Yong, Tafadzwa Sibindi, Samantha Foo, Rahmat Dapari

**Affiliations:** 1 Discipline of Social and Administrative Pharmacy, School of Pharmaceutical Sciences, Universiti Sains Malaysia, Penang, Malaysia; 2 General Paediatrics and Paediatric Cardiology, KPJ Selangor Specialist Hospital, Shah Alam, Selangor, Malaysia; 3 Medical Affairs, Takeda Malaysia Sdn. Bhd., Petaling Jaya, Selangor, Malaysia; 4 Department of Community Health, Faculty of Medicine and Health Sciences, Universiti Putra Malaysia, Serdang, Selangor, Malaysia

**Keywords:** attitude, dengue, knowledge, Malaysia, practice, vaccine

## Abstract

**Objectives:**

Malaysia continues to experience a high dengue burden, despite long-standing national education and control efforts. This study aimed to examine public Knowledge, Attitudes, and Practices related to dengue prevention, with a focus on informing more targeted education, vector control, and vaccination strategies.

**Methods:**

This study analysed Malaysia-specific data from the publication Knowledge, Attitudes and Practices toward Dengue Fever, Vector Control, and Vaccine Acceptance Among the General Population in Countries from Latin America and Asia-Pacific: A Cross-Sectional Study (Growth and Emerging Markets Knowledge, Attitude and Practice [GEMKAP]) conducted between September and October 2022. A total of 400 adults aged 18–60 from Malaysia completed the online questionnaire, which assessed dengue-related knowledge, attitudes, practices, and vaccine willingness.

**Results:**

Awareness of dengue transmission was high, however, misconceptions about reinfection risks, availability of a cure, and vaccine safety remain. Knowledge score (46%) aligned with global and Asia-Pacific averages, while Attitude (61%) and Practice (46%) scores were slightly lower than global benchmarks (66 and 48%, respectively). Although most respondents perceived dengue as a serious threat and reported removing stagnant water as a preventive measure, more intensive actions were less commonly practised. A total of 41% expressed high willingness to receive a dengue vaccine, as measured before a dengue vaccine TAK-003 (Qdenga) became available in Malaysia. Key barriers included concerns about side effects and insufficient information. Subgroup analysis showed higher willingness to vaccinate among respondents with prior dengue exposure, strong perceptions of disease severity, a generally pro-vaccine stance, and prior COVID-19 vaccination. While healthcare professionals were widely trusted sources of dengue information, this did not translate into higher vaccine acceptance. In addition, 40% of Malaysian respondents supported a multi-pronged dengue prevention strategy incorporating vaccination, education, and vector control.

**Conclusion:**

Future prevention efforts should prioritise coordinated messaging led by the Ministry of Health, through a multi-stakeholder task force involving healthcare professionals and community leaders. Efforts should focus on addressing misconceptions on dengue and vaccination, strengthening communication through trusted channels, and promoting more consistent adoption of vector control practices. Improving access to clear and reliable information on dengue vaccines will be critical to reducing hesitancy and supporting uptake.

## 1 Introduction

Dengue remains a persistent and escalating public health threat in Malaysia. The country’s tropical climate, characterised by sustained heat and humidity, creates favourable conditions for *Aedes* mosquito breeding, while rapid urbanisation and high population density in endemic regions exacerbate transmission risks. Malaysia continues to experience seasonal dengue outbreaks, contributing to increased hospitalisation rates and placing a significant strain on the national healthcare system (1).

Dengue incidence in Malaysia has fluctuated in recent years, with 122,423 cases reported in 2024 (2). More recently, reported cases declined to 48,766 as of November 2025, representing an approximate 58.6% decrease compared to 2024 (3). Despite this reduction, dengue continues to pose a significant public health burden, with persistent transmission across endemic areas. The highest burden was observed in Selangor and Kuala Lumpur. These highly urbanised areas have dense populations that exacerbate *Aedes* mosquito proliferation and virus transmission. Although most cases are mild, severe clinical presentations such as dengue haemorrhagic fever and dengue shock syndrome continue to pose serious public health risks. These conditions are associated with high morbidity and potential fatality. Dengue-related mortality has also increased significantly, rising by 78.6% from 2022 to 2023, followed by a further 17% increase in 2024 (2). In response, Malaysia has shifted from a vector control-centric approach to a more integrated dengue prevention strategy. The National Dengue Prevention and Control Strategic Plan (2022–2026) outlines a multipronged framework for dengue prevention and control. It strengthens entomological surveillance, enhances community-based vector control, and improves clinical management capacities (4). These efforts are complemented by the Wolbachia Malaysia project, an innovative intervention that releases Wolbachia-infected mosquitoes to biologically suppress vector populations. Initially launched as a pilot in selected hotspots, the program has since been scaled into a national initiative by Malaysian health authorities following successful field trials using the *Wolbachia Aedes aegypti* strain B (wAlbB), which showed sharp declines in dengue incidence (5). This national strategy is closely aligned with global recommendations from the World Health Organisation (WHO), which advocates for integrated dengue management as part of its Global Strategy for Dengue Prevention and Control. The WHO emphasises the need for comprehensive approaches that combine surveillance, case management, vector control, and vaccination to achieve zero dengue-related fatalities by 2030 (6).

In Malaysia, public practices related to dengue prevention remain inconsistent, suggesting underlying gaps in knowledge and attitudes. While some practices, such as clearing stagnant water, are commonly adopted, others involving the use of larvicides or protective clothing are less consistently practised, often due to misinformation or low perceived risk (7). Vaccination represents a promising yet underutilised component of Malaysia’s dengue control strategy. In WHO’s 2024 position paper, dengue vaccination was reaffirmed as an important complement to traditional prevention methods, especially in hyperendemic regions (8). Two vaccines, Dengvaxia and TAK-003 (Qdenga), have received WHO recommendations (8); however, their use differs globally and within Malaysia. Qdenga, a live attenuated tetravalent vaccine, received renewal of conditional approval from Malaysia’s Drug Control Authority (DCA) in February 2026 for use in individuals aged four and above, although its use remains subject to ongoing regulatory evaluation, based on official regulatory correspondence (Drug Control Authority Malaysia, unpublished; on file with Takeda Pharmaceutical Company, February 2026). In contrast, Dengvaxia received conditional registration in 2017 for use only within a Phase IV study and was not accessible to the general public (9).

Given the limitations of vector control alone, integrating vaccination into dengue prevention strategies is essential to reduce transmission and improve population-level protection. A more targeted and evidence-based approach requires a clearer understanding of how public knowledge, attitudes, and practices influence dengue prevention behaviours and vaccine acceptance in Malaysia. This manuscript presents a Malaysia-specific analysis of data from the 2023 multi-country Growth and Emerging Markets Knowledge, Attitude and Practice (GEMKAP) study, which examined public perceptions and behaviours related to dengue, vector control, and vaccination across Argentina, Brazil, Colombia, Mexico, Indonesia, Malaysia, and Singapore (6). While the parent GEMKAP study reported cross-country findings, it did not provide an in-depth interpretation of the Malaysian findings within the country’s epidemiological, policy, and healthcare context. Given Malaysia’s evolving dengue prevention strategy and vaccine landscape, a dedicated country-level analysis is warranted to translate these findings into Malaysia-specific public health recommendations for education, stakeholder engagement, and future vaccine implementation. Accordingly, this study aims to assess behavioural drivers and barriers to dengue prevention, as well as identify trusted information sources and communication channels among Malaysian adults based on data collected in 2022. These findings provide context-specific evidence to support the development of tailored public health messaging and more comprehensive, coordinated dengue prevention strategies in Malaysia.

## 2 Methods

### 2.1 Study design

The present study is a secondary analysis of the Malaysian subset of data collected by the multi-country GEMKAP study, which assessed knowledge, attitudes, and practices (KAP) related to dengue fever, vector control, and vaccination. The GEMKAP study was a quantitative, cross-sectional online survey conducted between September and October 2022. The study followed the Checklist for Reporting Results of Internet E-Surveys (CHERRIES) to ensure the quality and integrity of internet-based survey methodologies (10). To generate Malaysia-specific insights, only Malaysia data from the GEMKAP study was extracted, and to ensure contextual relevance and accuracy of the insights, three local infectious diseases experts, including a paediatrician, public health medicine specialist, and pharmacoeconomist (KPJ Selangor Specialist Hospital, Universiti Putra Malaysia, Universiti Sains Malaysia), were consulted.

### 2.2 Respondents

Prospective respondents were invited to join the survey through email outreach conducted via the mailing lists of established general-purpose online panel providers. A total of 400 adult respondents from Malaysia completed the online survey, which is sufficient to achieve national representativeness with a 95% confidence level and a 5% margin of error for descriptive analysis. All respondents took part in the survey voluntarily and provided electronic consent prior to participation. Respondents received an incentive upon successful completion of the survey.

Eligible respondents were between 18 (legal age in Malaysia) and 60 years old. The upper age limit of 60 was chosen for two primary reasons. First, dengue tends to affect children and younger adults more frequently in endemic settings such as Malaysia, making insights from these groups particularly relevant for prevention and control strategies. Second, restricting the sample to individuals aged 60 and below helped reduce disparities in digital literacy, thereby minimising potential selection bias. Quota sampling was used to ensure the sample was nationally representative of Malaysia’s adult population, with quotas defined by age, geographic region, and household income based on national census data and publicly available sources.

### 2.3 Survey development

The GEMKAP survey was developed through a review of existing published dengue KAP studies. The survey captured information aligned with the KAP framework, covering numerous domains: understanding of dengue and its prevention, perceptions of infection risk and views on the effectiveness of vector control, individual and community-level prevention behaviours, awareness and attitudes toward dengue vaccination and rollout, practice methods of dengue prevention and preferred sources of health information. Two cognitive interviews were conducted in Malaysia to validate and refine the questionnaire’s clarity, comprehension, cultural relevance, and response logic. The final survey included 35 questions, was administered in Malay, Chinese and English, and took approximately 30 min to complete. The questionnaire was translated from English into Malay and Chinese to ensure broader accessibility. Responses were collected in various formats, including binary (true/false), Likert scale, multiple choice, and open-ended questions. Each item was mapped to a corresponding KAP subdomain.

To uphold data quality, several data validation measures were embedded in the online survey. These included mandatory fields, Internet Protocol (IP) address checks, identity confirmation, digital fingerprinting, and engagement metrics to flag irregular or inattentive response patterns. Respondents were only permitted to complete the survey once, and duplicate entries were removed. A post-survey data cleaning process was conducted to filter out low-quality responses, including straight-line responses on multiple-choice questions, unusual response patterns and timestamps, and inconsistent or nonsensical open-text answers.

### 2.4 Covariates and outcomes

Sociodemographic and baseline characteristics collected included gender, age, household size, ethnicity, religion, region of residence, highest level of education, and household income. Additional baseline data captured respondents’ prior experience with dengue, their perceived level of dengue risk (low, moderate, or high), and their vaccination history for dengue, COVID-19, and influenza. All responses were self-reported.

The study’s primary outcome was willingness to receive the dengue vaccine, assessed using the Juster Scale, an 11-point scale ranging from 0 (no chance) to 10 (certain). Scores were categorised into three levels: high (8–10), moderate (4–7), and low (0–3) to facilitate interpretation of willingness to vaccinate, consistent with the categorisation applied in the GEMKAP study (6).

Secondary outcomes assessed respondents’ KAP related to dengue infection, symptoms, prevention methods, and vaccines. Each survey question was mapped to a specific KAP domain, and composite scores were calculated and normalised on a 0–100% scale. A score of 80–100% was classified as high, 50–79% as moderate, and 49% or below as low, in line with scoring approaches used in prior KAP and dengue-related research (11, 12).

### 2.5 Data analysis

Malaysia-specific data from the GEMKAP study were analysed using R, version 4.2.1. Descriptive statistics were applied to report baseline characteristics, including sociodemographic attributes. Categorical variables were reported as counts and percentages, while continuous variables were presented as means and standard deviations. This descriptive approach enabled the identification of key trends, patterns, and distributions within the dataset. To examine differences in willingness to vaccinate across population subgroups, multivariate regression analysis was conducted using generalised linear models, which were selected to examine associations between respondent characteristics and willingness to vaccinate while allowing adjustment for multiple covariates. These subgroup comparisons included variables such as age, gender, income, education level, ethnicity, religion, dengue experience, perceived risk, vaccination history, and general vaccine attitudes. Subgroup analyses were also carried out for secondary outcomes, evaluating variations in KAP scores across the same covariates, including endemic versus non-endemic regions, defined based on national endemicity levels. Given the cross-sectional design and use of non-probability quota sampling, results from regression analyses are interpreted as associative rather than causal.

### 2.6 Ethics and data confidentiality

The GEMKAP study was reviewed and granted exemption status by the Pearl Institutional Review Board (study Number: 22-VIST-101), an independent board accredited by the Association for the Accreditation of Human Research Protection Program Inc. (AAHRPP), because it involved a minimal-risk, anonymous survey of adults (13). Prior to participation, all respondents provided electronic informed consent. The survey did not collect, store, or transmit any personally identifiable information. All data were anonymised and managed in accordance with local data protection regulations. Data were securely stored, accessed only with authorisation, analysed in aggregate form, and archived via a permission-based system.

## 3 Results

**Figure 1:**
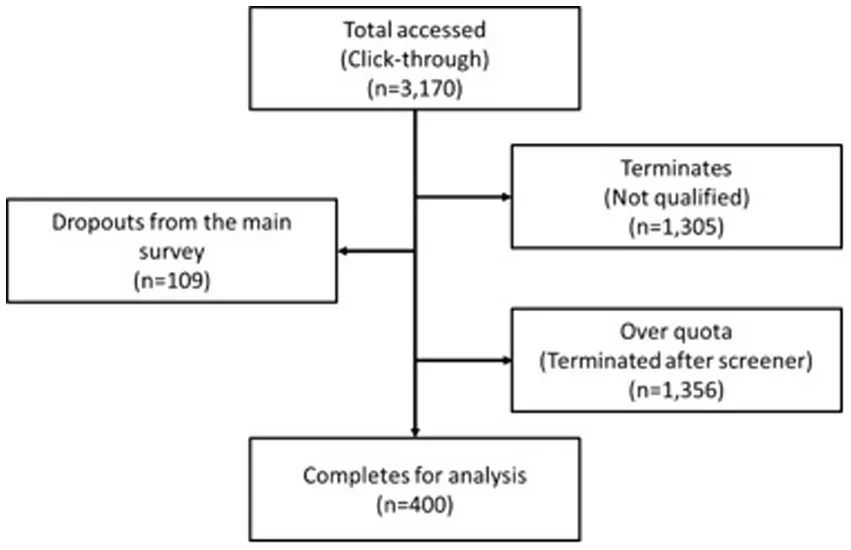
Response rate for the Malaysian population.

A total of 400 Malaysian adults aged 18–60 years participated in the study. The survey completion and inclusion process is summarised in Figure 1, which outlines the respondent flow from initial recruitment to final sample inclusion.

Table 1 summarises the sociodemographic characteristics of the study population. Quotas were applied for age, household income, and region, based on national census data, to ensure representation across key demographic subgroups. The sample consisted of 54% female respondents. Nearly half (48%) lived in households of three to four members, and 50% reported having no children.

**Table 1:** Socio-demographic characteristics of study respondents in Malaysia.

Demographic	Sociodemographic	*N* (%)
Gender	Male	184 (46%)
	Female	216 (54%)
Age (years)	18–30 years	161 (40%)
	31–40 years	105 (26%)
	41–50 years	74 (19%)
	51–60 years	60 (15%)
Household size	Live alone	16 (4%)
	1–2 members	52 (13%)
	3–4 members	192 (48%)
	5–6 members	104 (26%)
	>6 members	36 (9%)
Family household: number of children	0	201 (50%)
	1–2	152 (38%)
	3–4	41 (10%)
	>4	6 (2%)
Ethnicity	Chinese	177 (44%)
	Indian	15 (4%)
	Malay	203 (51%)
	Others	5 (1%)
Religion	Christianity	65 (16%)
	Islam	173 (43%)
	Buddhism or Taoism	128 (32%)
	Hinduism	13 (3%)
	Others	3 (1%)
	No religion	18 (5%)
Education level	No formal education	0 (0%)
	Primary education	3 (1%)
	Secondary education	71 (18%)
	Tertiary education	294 (73%)
	Post-tertiary education	32 (8%)
Level of income	High (11,000+ MYR)	80 (20%)
	Medium (5,000–10,999 MYR)	160 (40%)
	Low (<5,000 MYR)	160 (40%)
Prior dengue infection^1^	Yes	106 (26%)
	No	294 (74%)
Vaccinated against COVID-19	Yes	386 (97%)
	No	14 (3%)
Vaccinated against influenza^2^	Yes	95 (24%)
	No	305 (76%)

1 Based on respondents’ perception, regardless of their actual dengue infection serostatus.

2 Based on respondents’ perception, regardless of their actual influenza vaccination status.

MYR, Malaysian Ringgit.

### 3.1 Knowledge, attitude and practice

#### 3.1.1 Knowledge

**Figure 2:**
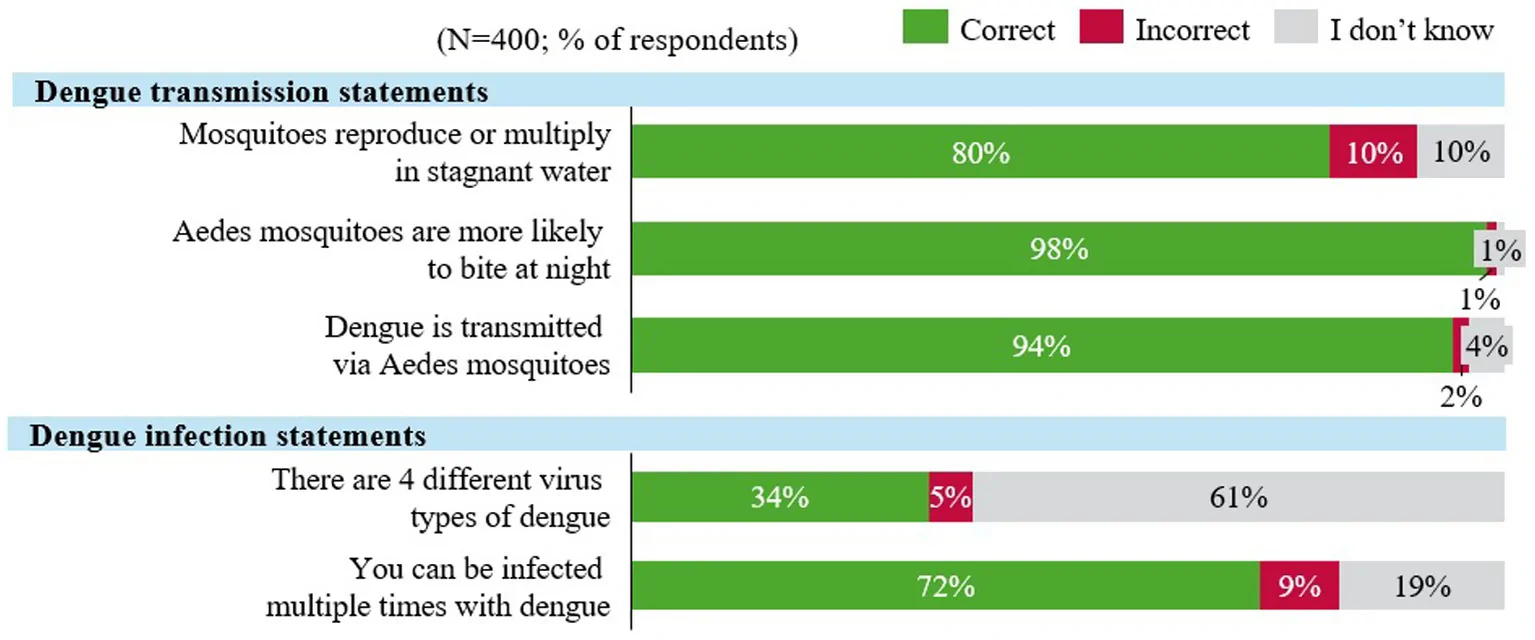
Knowledge on dengue transmission, infection and management.

Composite knowledge scores in Malaysia were moderate, averaging 46%. While most respondents were well-informed of basic dengue transmission pathways, a key misconception was identified, with 80% incorrectly believing that *Aedes* mosquitoes are more likely to bite at night. Additional knowledge gaps were observed in areas such as reinfection risk, virological complexity, and awareness of the dengue vaccine (Dengvaxia) available at the time of the survey. Notably, respondents with higher knowledge scores tended to engage in more intensive vector control practices (Figure 2).

**Figure 3:**
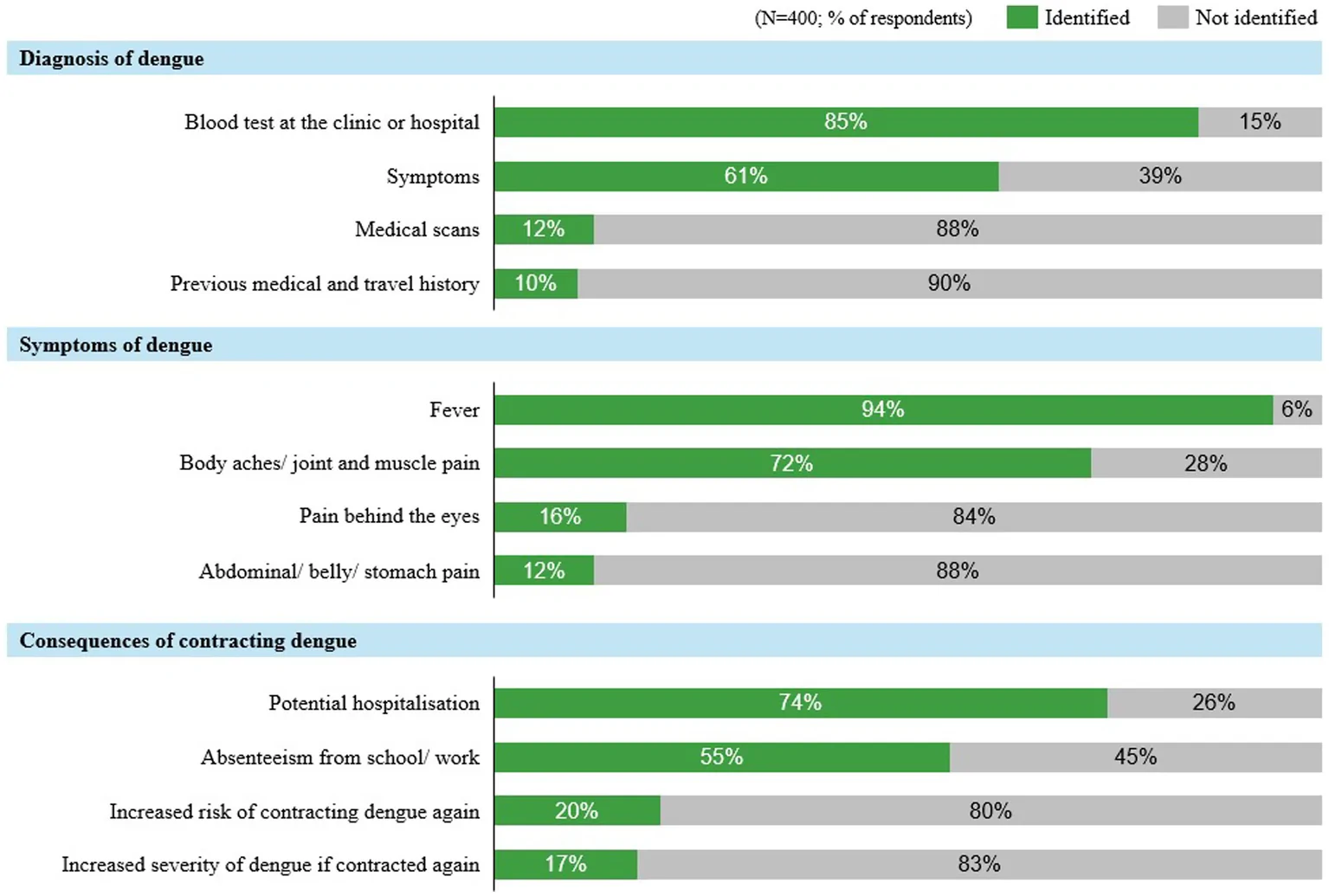
Knowledge on symptoms and consequences of dengue infection.

In terms of dengue symptom recognition, most respondents demonstrated good awareness of common signs such as fever and body aches. However, recognition of less typical symptoms remained low, with fewer than half identifying abdominal pain and pain behind the eyes as signs of dengue infection (Figure 3). The majority (85%) correctly identified that dengue is diagnosed through blood tests at clinics or hospitals. However, nearly two-thirds (61%) incorrectly reported that dengue could be diagnosed based on symptoms alone.

#### 3.1.2 Attitude

**Figure 4:**
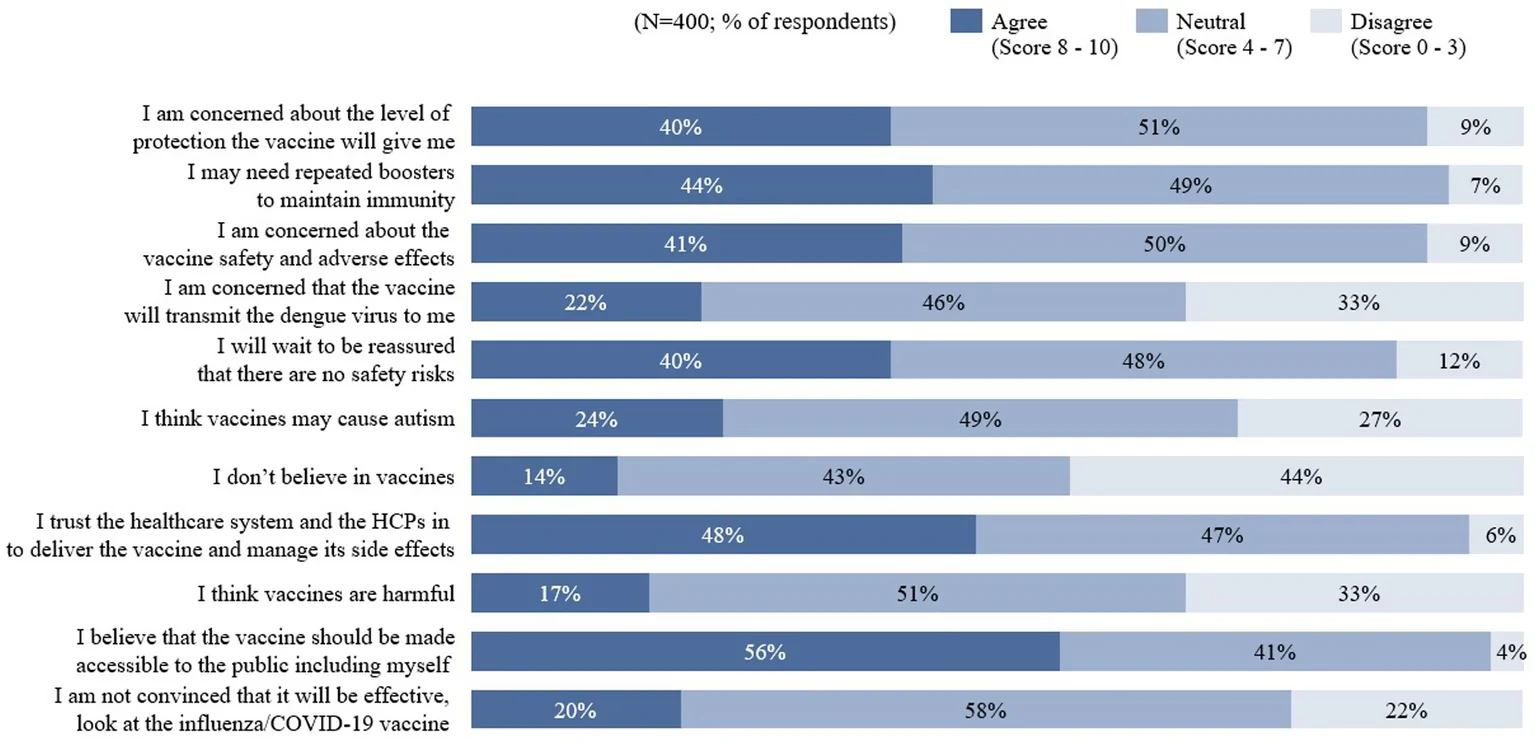
Attitudes regarding dengue vaccines in Malaysia.

The composite attitude score among Malaysian respondents was 61%. As shown in Figure 4, responses reflected moderate levels of vaccine confidence and considerable ambivalence toward dengue control measures.

Trust in vaccine safety and effectiveness was limited, likely reflecting the lack of publicly available dengue vaccines at the time of the survey. As dengue vaccines were not accessible to the general public in Malaysia (9), respondents’ views reflected hypothetical perceptions of dengue vaccination rather than experience with an available vaccine. Only 46% of respondents expressed pro-vaccination sentiments, while 34% believed vaccines are primarily for children, the older adults, and other vulnerable populations. Although doctors were the most trusted source of vaccine information (90%), this did not consistently translate into acceptance. Only 55% of respondents reported trusting doctors’ vaccination recommendations, and 56% believed that all vaccines approved by the Ministry of Health (MOH) are medically safe. Concerns over vaccine safety, potential adverse effects, and the possibility of transmission from the vaccine itself were frequently cited.

More broadly, Malaysians expressed less optimism about dengue prevention than respondents from other regions. There was higher agreement with statements reflecting fatalism, including “there is nothing we can do to prevent dengue” and “we just have to accept it,” with mean agreement scores of 2.9 and 4.2 out of 10, respectively. Additionally, perceptions that the media exaggerated the threat of dengue were more common in Malaysia (mean agreement score of 4.0).

Despite these concerns, confidence in the government’s dengue response and preparedness was relatively strong, with ratings of 6.4 and 5.9 out of 10, respectively. Notably, respondents with higher perceived risk of dengue and stronger trust in vaccine efficacy were more likely to be willing to vaccinate, underscoring the importance of addressing attitudinal barriers to support vaccine uptake.

#### 3.1.3 Practice

**Figure 5:**
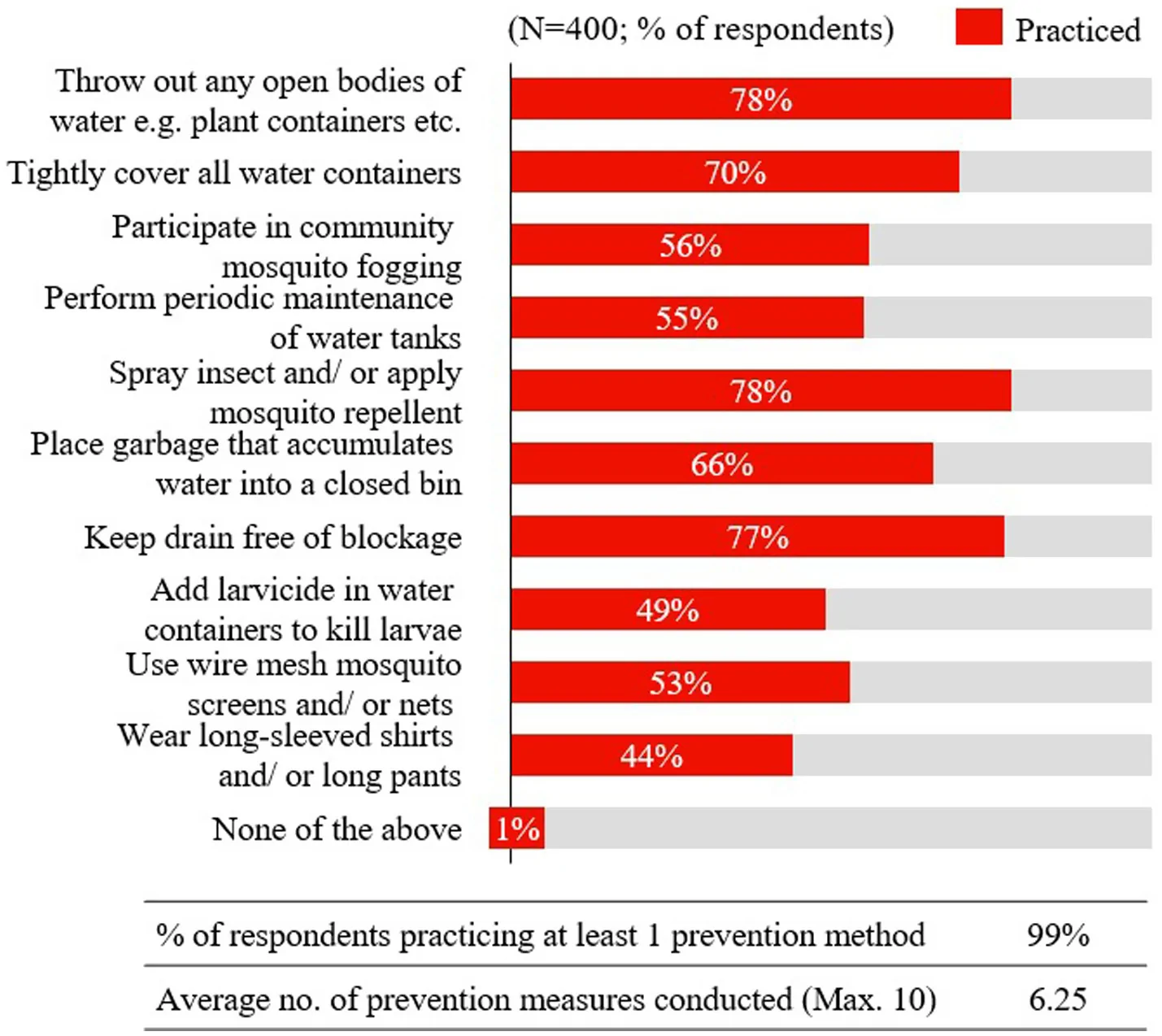
Prevention measures practised in Malaysia.

Practice scores in Malaysia averaged 46%. On average, respondents reported practising 6.3 out of 10 dengue prevention behaviours, slightly higher than the global average of 5.8. Nearly all respondents (99%) reported undertaking at least one preventive measure (Figure 5).

The most commonly adopted measures included disposing of open water sources (78%), using mosquito repellent or spray (78%), and keeping drains clear of blockages (77%). Other common practices included placing garbage in closed bins (66%) and tightly covering water containers (70%).

Less frequently reported behaviours included participating in community mosquito fogging (56%), performing periodic maintenance of water tanks (55%), and using wire mesh mosquito screens or nets (53%). The least practised methods were adding larvicide to water containers (49%) and wearing long-sleeved shirts or pants (44%).

Although a relatively high number of preventive behaviours were reported, many were not practised with the consistency recommended by international guidelines, such as those from the US Centers for Disease Control and Prevention (CDC). While certain actions, such as water disposal and repellent use, were performed regularly, others, such as participation in fogging activities and wearing protective clothing, were more sporadic.

A positive association was observed between knowledge and practice scores. Practice scores were also positively associated with increased perceived risk of dengue and willingness to vaccinate. These findings suggest that strengthening public understanding could help promote more consistent adoption of preventive behaviours.

### 3.2 Willingness to vaccinate

Willingness to receive the dengue vaccine was mixed among Malaysian respondents (Table 2). Overall, 41% of Malaysian respondents expressed a high willingness to vaccinate (score 8–10), followed by 52% of respondents who expressed moderate willingness (score 4–7), and 7% reported low willingness to vaccinate (score 0–3). Notably, high willingness remained unchanged at 41% even when the vaccine was recommended by a healthcare professional.

**Table 2:** Individual willingness to vaccinate against dengue in Malaysia by socio-demographic sub-groups (*N* = 400).

Demographic	Sociodemographic	Base	Mean	Standard Deviation	Willingness to vaccinate against dengue (*N*, %)		
					High willingness	Moderate willingness	Low willingness
Full study population	Malaysia	400	6.79	2.0	164 (41%)	208 (52%)	28 (7%)
	Malaysia (HCP recommended)	400	6.76	2.1	164 (41%)	204 (51%)	32 (8%)
Gender	Male	184	7.0	2.1	180 (45%)	193 (48%)	26 (7%)
	Female	216	6.7	2.0	144 (36%)	225 (56%)	30 (8%)
Age (years)	18–30	161	6.8	1.8	139 (35%)	243 (61%)	17 (4%)
	31–40	105	7.1	1.9	179 (45%)	202 (50%)	19 (5%)
	41–50	74	6.9	2.2	178 (45%)	189 (47%)	32 (8%)
	51–60	160	6.3	2.6	167 (42%)	167 (42%)	67 (16%)
Family household: number of children	0	201	6.7	2.0	145 (36%)	227 (57%)	28 (7%)
	1–2	152	7.0	2.0	187 (47%)	187 (47%)	26 (6%)
	3–4	41	6.7	2.1	146 (37%)	224 (56%)	29 (7%)
	>4	6	6.5	2.7	133 (33%)	200 (50%)	67 (17%)
Religion	Christianity	65	6.7	1.7	154 (39%)	234 (58%)	12 (3%)
	Islam	173	7.0	2.2	187 (47%)	180 (45%)	32 (8%)
	Buddhism or Taoism	128	6.6	1.8	116 (29%)	256 (64%)	256 (7%)
	Hinduism	13	8.4	1.5	308 (77%)	92 (23%)	–
	Others	3	3.3	4.2	133 (33%)	–	267 (67%)
	No religion	18	6.4	2.3	156 (39%)	222 (56%)	22 (5%)
Education level	No formal education	0	0	0	–	–	–
	Primary education	3	8	2	267 (67%)	133 (33%)	–
	Secondary education	71	6.7	2.2	158 (40%)	208 (52%)	34 (8%)
	Tertiary education	294	6.8	1.9	163 (41%)	212 (53%)	24 (6%)
	Post–tertiary education	32	6.7	2.5	138 (34%)	213 (53%)	50 (13%)
Level of income	High	160	6.8	2.1	163 (41%)	213 (53%)	25 (6%)
	Medium	160	6.9	1.9	158 (39%)	223 (56%)	20 (5%)
	Low	80	6.6	2.3	165 (41%)	185 (46%)	50 (13%)
Prior dengue infection	Yes	106	6.8	1.7	132 (33%)	253 (63%)	15 (4%)
	No	294	6.8	2.1	176 (43%)	172 (49%)	26 (8%)
Vaccinated against COVID-19	Yes	386	6.9	2.0	165 (41%)	209 (52%)	26 (7%)
	No	14	4.7	2.5	57 (14%)	257 (64%)	86 (22%)
Vaccinated against influenza	Yes	95	7.1	1.8	186 (46%)	202 (51%)	13 (3%)
	No	305	6.7	2.1	153 (39%)	213 (53%)	33 (8%)
Level of perceived risk	Very high risk	98	7.5	2.0	224 (56%)	159 (40%)	16 (4%)
	High risk	130	6.8	1.9	163 (41%)	203 (51%)	34 (8%)
	Medium risk	134	6.5	1.9	122 (30%)	251 (63%)	27 (7%)
	Low risk	20	6.2	2.5	140 (35%)	220 (55%)	40 (10%)
	Very low risk	11	6.1	1.9	73 (18%)	291 (73%)	36 (9%)
	No risk	7	6.7	2.7	171 (43%)	171 (43%)	57 (14%)
Opinion toward vaccine	Very useful	164	7.7	1.8	251(63%)	139 (35%)	10 (2%)
	Useful	152	6.7	1.7	134 (33%)	242 (61%)	24 (6%)
	Somewhat useful	64	5.5	1.8	44 (11%)	306 (77%)	50 (12%)
	Slightly useful	16	4.6	1.8	–	300 (75%)	100 (25%)
	Not useful	4	3	1.6	–	100 (25%)	300 (75%)
	Not useful at all	0	0	0	–	–	–

Subgroup analysis showed higher willingness to vaccinate among individuals with prior dengue infections, those with a strong perceived risk of dengue, and those who held positive views about vaccines in general. For instance, 52% of respondents who had previously contracted dengue reported high willingness to vaccinate, compared with 39% among those without prior infection. Similarly, 56% of respondents who perceived dengue risk as “very high” expressed high willingness to vaccinate, versus only 17% among those who perceived dengue as “very low” risk. Vaccine sentiment played a significant role as 63% of those who considered vaccines “very useful” reported high willingness to vaccinate, compared to just 11% among those who viewed vaccines as only “somewhat useful.”

Multivariate regression analysis showed that gender and ethnicity were significantly associated with willingness to vaccinate (*p* < 0.05). Female respondents were less likely to express willingness to vaccinate, while respondents of Indian and Malay ethnicity were more likely to express willingness to vaccinate against dengue relative to other sociodemographic sub-groups. Full multivariable regression results are provided in Supplementary Table S1.

**Figure 6:**
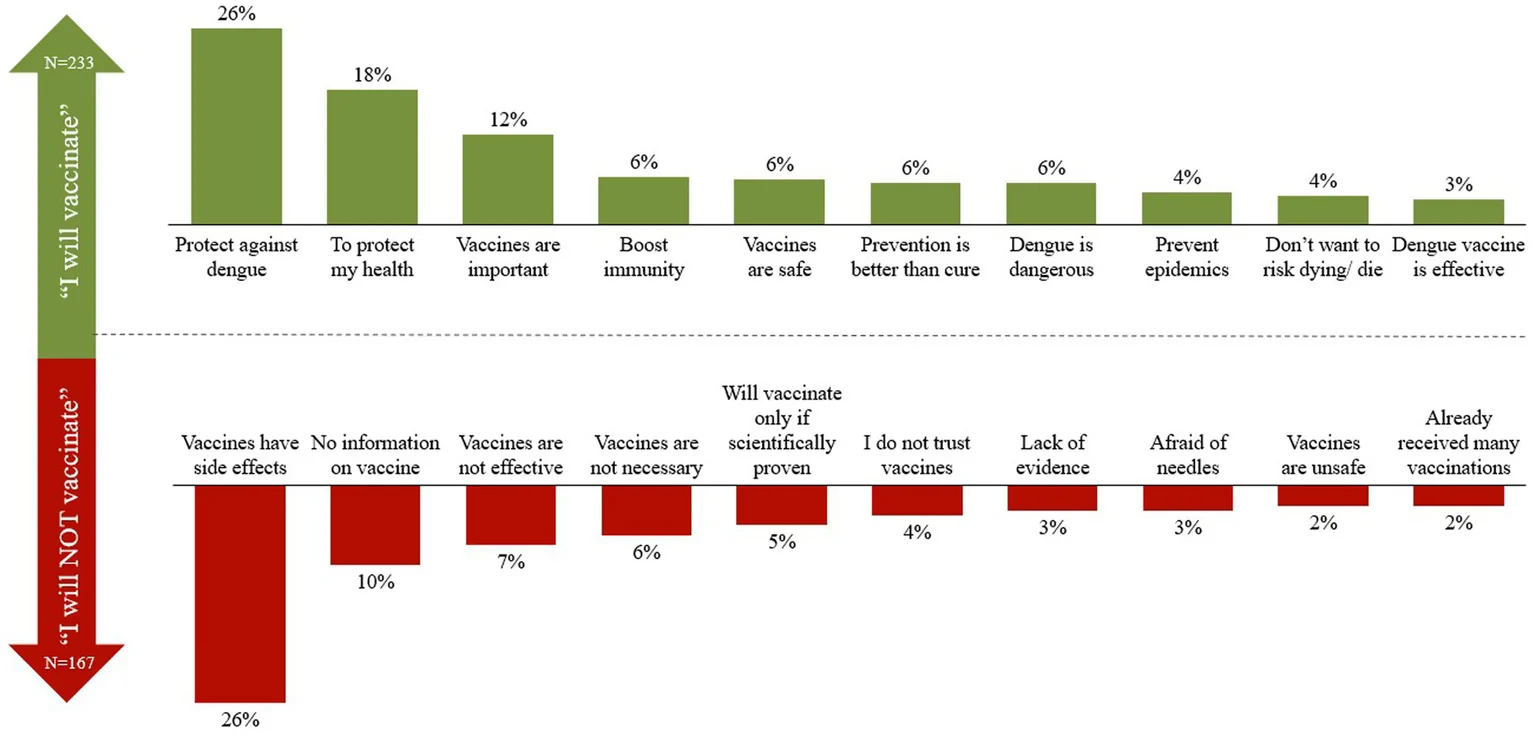
Reasons for willingness or unwillingness to vaccinate.

Among respondents willing to vaccinate, the leading reasons included protection against dengue (26%), protecting personal health (18%), and belief in the importance of vaccines (12%). In contrast, vaccine hesitancy was driven primarily by concerns over side effects (26%), lack of information (10%), and doubts about vaccine efficacy (7%) (Figure 6).

### 3.3 Preferences in communication and dengue management

Doctors and other healthcare professionals (HCPs) were the most trusted source for health-related information (90%). Other trusted sources included pharmacists (52%) and nurses or paramedics (44%), with the government also seen as a key factor in health communication (39%).

When asked about preferred channels for health-related information, respondents indicated strong reliance on digital platforms. Search engines (85%) and social media (75%) were the most frequently used channels for searching for healthcare information, followed by government or health agency websites (54%).

**Figure 7:**
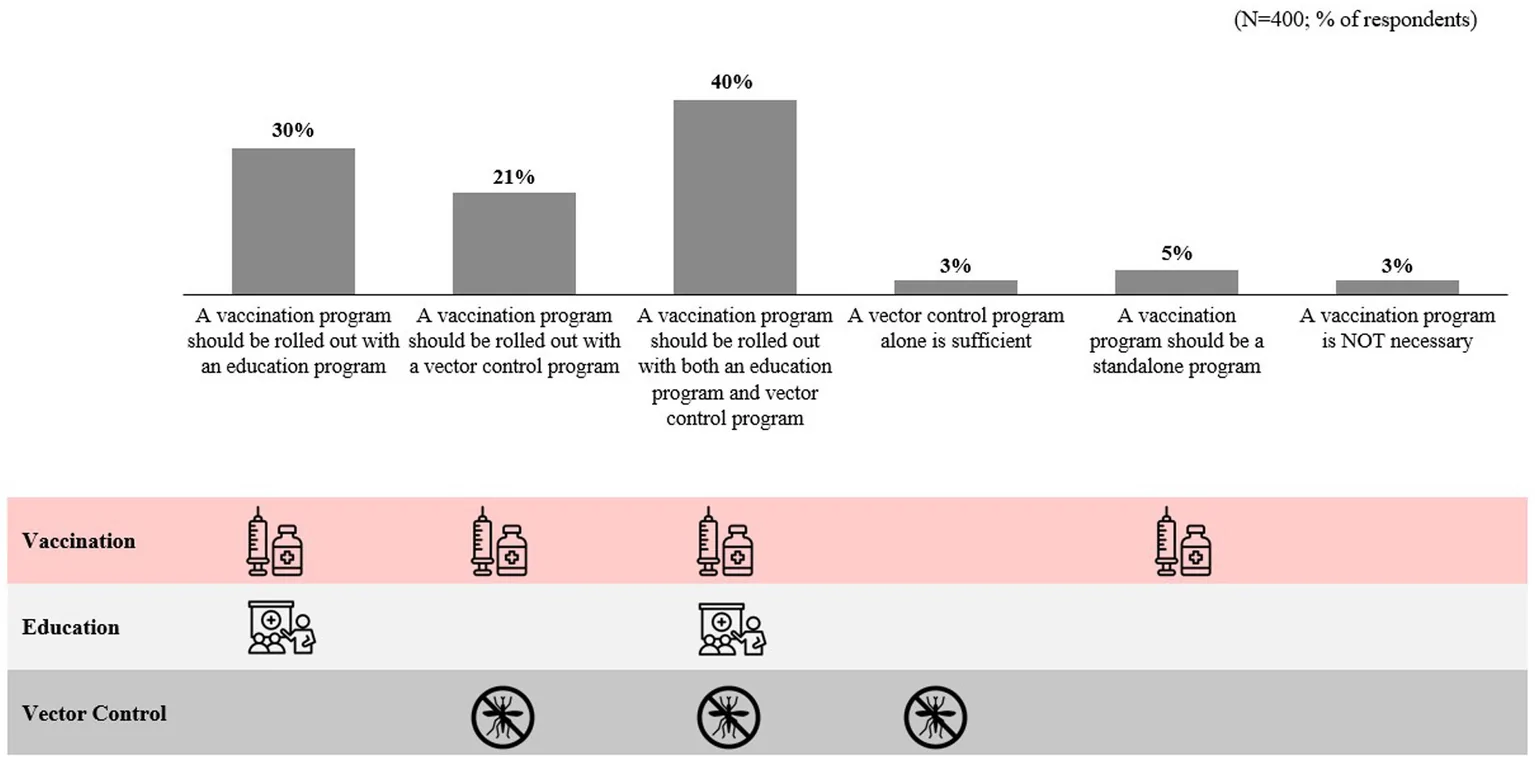
Preference for approaches toward nationwide dengue management.

When asked about their preferred strategy for dengue prevention, 40% of Malaysian respondents selected a combined strategy incorporating vaccination, education, and vector control as their preferred model (Figure 7). In contrast, support for single-strategy approaches was minimal.

## 4 Discussion

This cross-sectional study, conducted in 2022, prior to the introduction of TAK-003, the first and only dengue vaccine approved for public use in Malaysia in 2024, explored the Knowledge, Attitudes, and Practices of Malaysian adults toward dengue prevention and vaccination, in the context of a rising national disease burden. This Malaysia-specific analysis extends the findings of the multi-country GEMKAP study by translating cross-country survey findings into context-specific recommendations to inform dengue education, stakeholder communication, and future vaccination strategies in Malaysia. While respondents demonstrated foundational awareness of dengue and engaged in some prevention behaviours, significant gaps in knowledge and attitudes, particularly regarding vaccine acceptance, persisted. Overall, the findings highlight several priorities for Malaysia’s evolving dengue prevention strategy, including addressing persistent misconceptions about dengue, strengthening public confidence in vaccination, and reinforcing consistent preventive behaviours through coordinated stakeholder engagement. Given Malaysia’s transition toward a more integrated dengue prevention strategy, these findings support the need for locally tailored interventions that complement existing vector control measures with targeted education and future vaccination initiatives.

### 4.1 Addressing KAP gaps in dengue disease and prevention through a multi-pronged approach

Findings from the Malaysian cohort highlight several key areas of concern across the KAP spectrum. Composite knowledge scores in Malaysia averaged 46%, broadly comparable to Asia-Pacific (APAC) (47%) and global (48%) levels, indicating that despite longstanding national awareness initiatives, important misconceptions about dengue transmission, reinfection, treatment, and vaccination persist. Respondents demonstrated a general awareness of dengue transmission, such as recognising that dengue is transmitted by *Aedes* mosquitoes and the risk of multiple dengue infections. This baseline awareness may reflect the impact of long-standing public health efforts, including national campaigns, school-based education, and community clean-up (gotong-royong) activities, as well as MOH-led awareness efforts through mass media and the introduction of digital tools such as dengue hotspot tracking apps and SMS alerts (14–16).

However, Malaysian respondents had misconceptions about the number of dengue virus types, the risk of reinfection, and the availability of dengue treatment and vaccines. These findings echo regional trends identified in other Asian contexts, where high baseline awareness of dengue does not always translate into accurate knowledge or consistent preventive behaviour. A recent systematic review on KAP levels of dengue in Asia noted that knowledge of dengue was generally adequate across many studies, but attitude and practice scores often lagged, highlighting a recurring disconnect between understanding and action (17). These findings underscore the need for more targeted health education efforts that go beyond surface-level awareness to address persistent misinformation and improve overall dengue literacy.

Education efforts should focus on clarifying facts that are frequently misunderstood by the public, such as the risk of reinfection with different dengue serotypes, the potentially severe outcomes of secondary infections, and the current lack of a medicinal cure. Misconceptions about vaccines, particularly concerns around safety and efficacy, must also be proactively addressed through transparent, evidence-based communication.

To enhance public trust and credibility, these messages should be delivered by highly trusted messengers such as doctors, pharmacists, and government agencies. However, health campaigns should also take care to avoid overly dramatic or alarmist tones, which may inadvertently heighten scepticism rather than build confidence.

Attitudes toward dengue prevention were also lower in Malaysia (61%) than regional (63%) and global (66%) benchmarks, with evident scepticism toward vaccine safety and efficacy. This lack of trust may be associated with Malaysia’s lower levels of willingness to vaccinate against dengue, despite high endemicity. Addressing such concerns will require deliberate and transparent communication strategies, alongside greater involvement from the medical and scientific communities as trusted stakeholders to build public confidence in the safety and benefits of dengue vaccines. To help shift attitudes, community-led initiatives for high-risk groups can play a powerful role. Reinforcing the idea that dengue control is a shared responsibility between individuals and public authorities may foster greater ownership.

In terms of prevention practices, Malaysian respondents scored 46% on average, similar to APAC (47%) and global (48%) levels, but showed selective uptake of control measures. While most respondents reported eliminating open water sources and using mosquito repellent, the use of larvicides and wearing protective clothing was considerably lower than APAC and global averages. This pattern is consistent with the aforementioned hotspot study, which found that while over 80% of respondents practised basic dengue prevention such as removing stagnant water, fewer than 30% used mosquito nets or wore long-sleeved clothing (18). These selective practices suggest there is an inconsistent reach of community-level awareness initiatives such as recent hygiene campaigns led by the Malaysian Red Crescent and MOH in high-risk districts (19).

Taken together, the identified KAP gaps call for a multi-pronged dengue management program. Malaysians favour a comprehensive strategy that integrates health education, vector control, and vaccination. This approach is further supported by regional studies demonstrating that integrated interventions are more effective in driving sustained behaviour change than standalone efforts (17). Such a program should reinforce consistent adherence to proven vector control measures, strengthen community education, and improve access to and confidence in dengue vaccines as complementary components of an integrated dengue prevention strategy. Public health interventions should align with these preferences, delivering consistent messaging on the risks of dengue and the importance of prevention through credible channels.

### 4.2 Willingness to vaccinate

At the time of the study, no dengue vaccine was yet available for public use in Malaysia, and responses therefore reflected willingness to vaccinate with a hypothetical dengue vaccine. Malaysians demonstrated a lower willingness (41%) to receive a dengue vaccine compared to regional (52%) and global (53%) averages. A significant proportion of respondents reported adopting a “wait and see” approach, reflecting uncertainty about the vaccine’s safety, efficacy, or relevance to their health risk in the absence of local vaccine availability. This suggests that successful implementation of future dengue vaccination programmes will depend not only on vaccine availability but also on strengthening public confidence through clear communication, trusted recommendations, and transparent engagement around vaccine safety and effectiveness.

Interestingly, even among individuals who actively engaged in preventive behaviours, vaccine hesitancy remained prominent. This disconnect highlights that protective behaviours do not necessarily correlate with willingness to vaccinate, pointing to the need for tailored communication approaches. Perceived social approval, especially from family, peers, and respected authorities, has been identified as a key driver of willingness to vaccinate in Malaysia, reinforcing the value of coordinated, consistent messaging from multiple trusted sources (20).

In contrast to findings in other studies, HCP recommendations in Malaysia did not significantly improve vaccine acceptance, remaining at 41%. This may reflect both limited familiarity and underlying scepticism toward dengue vaccines at the time, as well as a lack of strong advocacy from medical professionals. As Malaysia prepares for broader implementation of dengue vaccination, strengthening HCP knowledge, confidence, and communication skills will be critical to ensure that vaccine recommendations are delivered consistently and effectively. In addition, structural barriers such as healthcare access, affordability, and socioeconomic circumstances may also influence vaccine uptake and should be considered when designing future dengue vaccination programmes. Trust in healthcare providers and public health institutions has been shown to significantly influence stakeholder acceptance of the dengue vaccine, suggesting that efforts to build institutional credibility and reassure the public may improve willingness to vaccinate (20). However, trust alone may be insufficient to drive uptake. These findings highlight the need for coordinated communication strategies that equip HCPs and public health authorities with clear, evidence-based messaging to translate confidence into vaccination decisions.

### 4.3 Multi-stakeholder collaboration

Effective dengue prevention in Malaysia hinges on coordinated efforts among key stakeholders. The MOH should take a central leadership role in aligning national strategies across vector control, health education, and vaccination. To ensure consistency in messaging across stakeholders with differing priorities, a dedicated multi-stakeholder health promotion task force, led by MOH, could be established to coordinate communication strategies and implementation efforts. To build public confidence, particularly among the 44% of Malaysian respondents uncertain about MOH-approved vaccines, clear, transparent communication from MOH is critical and should align with messaging from HCPs. Greater coordination with state and district-level authorities can ensure more locally tailored campaigns, supported by a formal framework for resource-sharing and unified monitoring. For example, digital and social media campaigns may be more appropriate in urban settings, whereas community outreach through local leaders, schools, and primary healthcare facilities may be more effective in rural communities. A national Call to Action, such as a designated dengue awareness week, could help unify efforts while allowing each state to lead context-specific activities.

HCPs, religious leaders, and community actors are also instrumental in shaping public attitudes. Clinicians can reinforce vaccine guidance by using culturally relevant language and providing individualised risk-based recommendations, especially for high-risk groups. Religious and community leaders, particularly within Muslim-majority communities, can enhance message credibility by addressing common misconceptions and affirming vaccine compatibility with faith values. Engagement with mosque leaders has proven especially important in countering misinformation and fostering trust among Muslim populations (21).

To maximise reach and effectiveness, communication should leverage trusted, culturally appropriate platforms. These include social media (e.g., TikTok, WhatsApp), radio, posters, and multilingual materials at healthcare and non-healthcare touchpoints like clinics, schools, and religious venues. Combining digital and traditional communication channels may help ensure information reaches populations with varying levels of digital literacy. The adoption of a Communication for Behavioural Impact (COMBI) approach by MOH and its partners could further strengthen these efforts by combining health education with targeted social mobilisation and marketing strategies to drive sustained behavioural change. COMBI’s structured planning process, from situation analysis to multi-channel delivery and community engagement, has been shown to strengthen community engagement and promote sustained uptake of vector control behaviours (22). This could similarly be applied to enhance vaccination and prevention initiatives in Malaysia (22). Empowering HCPs to serve as Digital Opinion Leaders may further increase public engagement and uptake, as Digital Opinion Leaders with medical credentials are more credible and effective than non-medical influencers in driving vaccine uptake and behaviour change (23).

### 4.4 Future research directions

To strengthen dengue vaccination and prevention strategies in Malaysia, future research should focus on context-specific behavioural and structural barriers. Implementation science frameworks, such as the consolidated framework for implementation research, can help assess factors influencing vaccine adoption, including individual behaviours, organisational readiness, and communication effectiveness (24).

Qualitative studies, such as KAP focus groups across different states, can reveal regional perceptions and inform targeted interventions. Research should also explore how structural barriers, including healthcare access, convenience, cost, and socioeconomic factors, influence dengue prevention behaviours and vaccine uptake. These insights can inform delivery models like mobile or school-based vaccination, especially in underserved areas. Malaysia can further build on successful community-led initiatives, such as the Integrated Dengue Education and Learning (iDEAL) module and *Aedes*Tech Mosquito Home System (AMHS) trial, which demonstrate the value of locally adapted, evidence-based strategies supported through continued MOH-academia collaboration (25).

Given that this survey was conducted prior to the introduction of TAK-003, the first dengue vaccine approved for public use in Malaysia, subsequent research could explore changes in public knowledge and attitudes toward the dengue vaccine. In particular, repeating the KAP study in the post-introduction period would provide insights into whether willingness to vaccinate has translated into observed vaccination uptake, and how vaccine confidence has evolved.

Lastly, further research is required to understand the association between willingness to vaccinate and sociodemographic subgroups, particularly gender and ethnicity, where females showed lower willingness and respondents from Indian and Malay ethnic groups showed higher willingness.

### 4.5 Limitations

This study provides timely insights into Malaysians’ knowledge, attitudes, and practices toward dengue prevention and vaccination. However, several limitations should be considered. First, as with all self-reported surveys, recall and social desirability bias may have influenced responses, although anonymity and the self-administered format likely reduced these effects. Second, while quota sampling accounted for key demographics, factors such as education and digital literacy were not explicitly included and may affect health knowledge and access. Third, recruitment through an online panel and the web-based survey format may have introduced selection bias by favouring individuals who were digitally literate and had internet access, potentially underrepresenting those with limited internet access, particularly in rural areas, where internet penetration in Malaysia is lower (90.3%) than in urban areas (98.8%) (26). A mixed-mode approach to improve representativeness in future research can be considered. Fourth, the study was restricted to adults aged 18–60 years; therefore, the findings may not be generalisable to older adults, who may have different health-seeking behaviours, communication preferences, and perceptions of dengue prevention and vaccination. Fifth, given the cross-sectional design, observed associations should be interpreted as non-causal. In addition, formal diagnostic assessment of regression model assumptions was not conducted; therefore, findings should be interpreted with appropriate caution. Finally, as a secondary analysis of the GEMKAP dataset, this study was limited to variables collected in the original survey. Consequently, additional Malaysia-specific factors that may influence dengue prevention behaviours and vaccine acceptance could not be examined. Furthermore, as the data were collected in late 2022, the findings reflect a single time point and may not capture subsequent changes in public perceptions following shifts in dengue incidence, policy developments, or vaccine availability. Repeated surveys across different time points could provide more dynamic insights to guide future interventions.

## 5 Conclusion

This study found that while dengue-related knowledge among Malaysians is moderate, critical gaps remain in public understanding, particularly around reinfection risks, treatment misconceptions, and vaccine awareness. Although many respondents reported engaging in preventive behaviours, adherence to key vector control practices was inconsistent, and vaccine hesitancy persists. To strengthen Malaysia’s dengue response, a coordinated approach integrating education, vector control, and vaccination is essential. Public health campaigns should prioritise clear, evidence-based messaging delivered by trusted stakeholders, while improving vaccine accessibility and affordability to encourage widespread uptake and long-term disease prevention.

## Data Availability

The original contributions presented in the study are included in the article/Supplementary material, further inquiries can be directed to the corresponding author.
