# Supplementary material for: Strengthening public understanding and prevention of dengue in Malaysia: insights and recommendations based on knowledge, attitudes, and practices toward vector control and vaccination
Source: Front Public Health. 2026 Aug 20;14:1807183. doi: 10.3389/fpubh.2026.1807183 (PMC13538089; doi:10.3389/fpubh.2026.1807183)
Supplement: Supplementary file 1 [file Table_1.DOCX]

**SUPPLEMENTARY FILE**

**Supplementary Table 1: Multivariate Regression Results on Population Subgroups with Willingness to Vaccinate against Dengue in Malaysia***

|  | **COEF** | **SE** | **P-value** |
| --- | --- | --- | --- |
| **Region (Endemic and Wolbachia cities)** |  |  |  |
| Non-Endemic Region | -0.300 | 0.273 | 0.274 |
| Non-Wolbachia cities | -0.108 | 0.246 | 0.662 |
| **Perceived Risk of Dengue** |  |  |  |
| Very low risk | 0.116 | 0.825 | 0.889 |
| Low risk | -0.787 | 0.754 | 0.297 |
| Medium risk | -0.809 | 0.660 | 0.222 |
| High risk | -0.704 | 0.666 | 0.291 |
| Very high risk | -0.656 | 0.675 | 0.332 |
| **Gender** |  |  |  |
| Female | -0.452 | 0.174 | 0.010 |
| **Age** |  |  |  |
| 31–40 years | 0.226 | 0.229 | 0.324 |
| 41–50 years | -0.198 | 0.259 | 0.446 |
| 51–60 years | -0.390 | 0.296 | 0.188 |
| **Household Size** |  |  |  |
| 1-2 members | 0.287 | 0.474 | 0.546 |
| 3-4 members | 0.206 | 0.431 | 0.634 |
| 5-6 members | 0.073 | 0.454 | 0.873 |
| >6 members | 0.389 | 0.510 | 0.446 |
| **Level of Education** |  |  |  |
| Primary education | 1.462 | 1.018 | 0.152 |
| Secondary education | 0.017 | 0.372 | 0.964 |
| Tertiary education | -0.038 | 0.326 | 0.908 |
| **Ethnicity** |  |  |  |
| Chinese | 1.375 | 0.934 | 0.142 |
| Indian | 2.356 | 1.154 | 0.042 |
| Malay | 2.005 | 0.920 | 0.030 |
| **Religion** |  |  |  |
| Buddhism | 0.438 | 0.444 | 0.325 |
| Christianity | 0.268 | 0.471 | 0.569 |
| Hinduism | 1.106 | 0.869 | 0.204 |
| Islam | -0.111 | 0.526 | 0.834 |
| Others | -2.222 | 1.218 | 0.069 |

COEF: Coefficient, SE: Standard Error

*Only regression coefficients for non-reference categories are presented. The reference category for each categorical variable was omitted because its regression coefficient is fixed at zero and serves as the comparator for all reported estimates.
